# Case Report: A personalized model of care for surgical resection of complex brain tumor with atypical language dominance

**DOI:** 10.3389/fnins.2025.1587594

**Published:** 2025-07-24

**Authors:** Natalie Sherry, Neslihan Nisa Gecici, Amelia Stepniak, Daniel Miller, Ahmed Habib, Ajay Niranjan, Jessica Barrios-Martinez, Fang-Cheng Yeh, Jeffrey Balzer, Pascal O. Zinn

**Affiliations:** ^1^Department of Neurological Surgery, University of Pittsburgh, Pittsburgh, PA, United States; ^2^Department of Neurosurgery, University of Pittsburgh Medical Center, Pittsburgh, PA, United States; ^3^UPMC Hillman Cancer Center, University of Pittsburgh Medical Center, Pittsburgh, PA, United States; ^4^University of Pittsburgh School of Medicine, Pittsburgh, PA, United States; ^5^Dietrich School of Arts and Sciences, University of Pittsburgh Medical Center, Pittsburgh, PA, United States

**Keywords:** brain tumor, language, atypical, neuropsychology, neurosurgery, case report, intraoperative mapping, right hemisphere

## Abstract

The gold standard for preserving language and cognitive function during complex brain tumor resection is direct electrical stimulation (DES) during awake craniotomy. DES is predominantly utilized for left hemisphere (LH) tumors, where language dominance is most common. However, atypical language dominance and functional reorganization due to tumor growth are increasingly recognized and can complicate preoperative planning. We present the novel case of a 58-year-old monolingual, English-speaking, strongly right-handed male with recurrent right temporal glioblastoma who exhibited right hemisphere (RH) language dominance based on multimodal preoperative evaluation. The patient had no known perinatal or neurodevelopmental history, and he had previously undergone tumor resection under general anesthesia, with no postoperative aphasia. An awake craniotomy with intraoperative mapping (IOM) was performed, which confirmed language representation in the right frontal and temporal lobes. This was further substantiated by neuropsychological testing (NPT), which revealed a decline in semantic language postoperatively. This case challenges the prevailing practice of limiting awake procedures to LH tumors and supports a personalized, multimodal approach to mapping eloquent cortex irrespective of tumor laterality to optimize surgical outcomes.

## 1 Introduction

Awake craniotomy with direct electrical stimulation (DES) is considered the gold standard for mapping and preserving language and other cognitive functions in the context of complex brain tumor resection (Young et al., 2021). The ultimate goal in the resection of a complex brain tumor is a supramaximal resection; however, the benefits of an extended resection are negated if the patient suffers from permanent neurological deficits, such as postoperative aphasia or sensorimotor impairment (Rahman et al., 2016). The majority of awake craniotomies are performed on patients with left hemisphere (LH) neoplasms, given the preponderance of individuals with LH dominance for language and the necessity of avoiding postoperative aphasia (Young et al., 2021; Knecht et al., 2000b). Approximately 90% of right-handers exhibit LH language dominance (Knecht et al., 2000a), but even among left-handers, ipsilateral language representation in the LH is common (Knecht et al., 2000b).

Historically, right hemisphere (RH) tumors have been resected under general anesthesia based on the assumption that the RH plays a minimal role in language. However, growing evidence suggests that atypical or bilateral language representation may be more common than previously believed (Knecht et al., 2003) and that the RH can subserve language and other eloquent functions in some individuals (Vilasboas et al., 2017). There is also the possibility of reorganization of functions in the circumstance of neurological insult or pathology (Duffau, 2006), such as tumor growth, resulting in the non-dominant hemisphere supporting aspects of language function. This interindividual variability in the organization of language challenges the routine use of general anesthesia for RH tumor resections (Tate et al., 2014), particularly in patients presenting with language symptoms and/or tumors located in eloquent regions (Vilasboas et al., 2017).

We present the case of a 58-year-old, strongly right-handed, monolingual English-speaking male with recurrent right temporal glioblastoma and new-onset speech disturbance. He did not demonstrate the usual risk factors associated with atypical language representation (i.e., mixed handedness, perinatal complications, or multilingualism) (Vilasboas et al., 2017) and he had undergone a prior resection under general anesthesia with no postoperative aphasia. Given the potential risk to the eloquent cortex, we undertook a conservative approach to comprehensively localize language function, and the patient ultimately required an awake craniotomy. This case highlights the importance of an individualized, multimodal evaluation guiding neurosurgical decision-making, even for RH tumors in patients without clear risk factors for atypical lateralization.

## 2 Case description

A 58-year-old right-handed, monolingual English-speaking male with a history of recurrent right temporal lobe glioblastoma (MGMT promoter methylated, TERT mutation positive) was on surveillance until he presented with an intermittent speech disturbance and severe headache. He had previously undergone a right-sided craniotomy for tumor resection at an outside institution 5 years prior, followed by adjuvant radiotherapy and chemotherapy, with his last chemotherapy treatment completed 4 years ago. His surveillance magnetic resonance imaging (MRI) scans remained stable until his recent presentation. In the month before presenting to our institution, he reported “slurred speech,” left-sided facial drooping, and intermittent twitching of his left upper and lower extremities.

**Figure 1:**
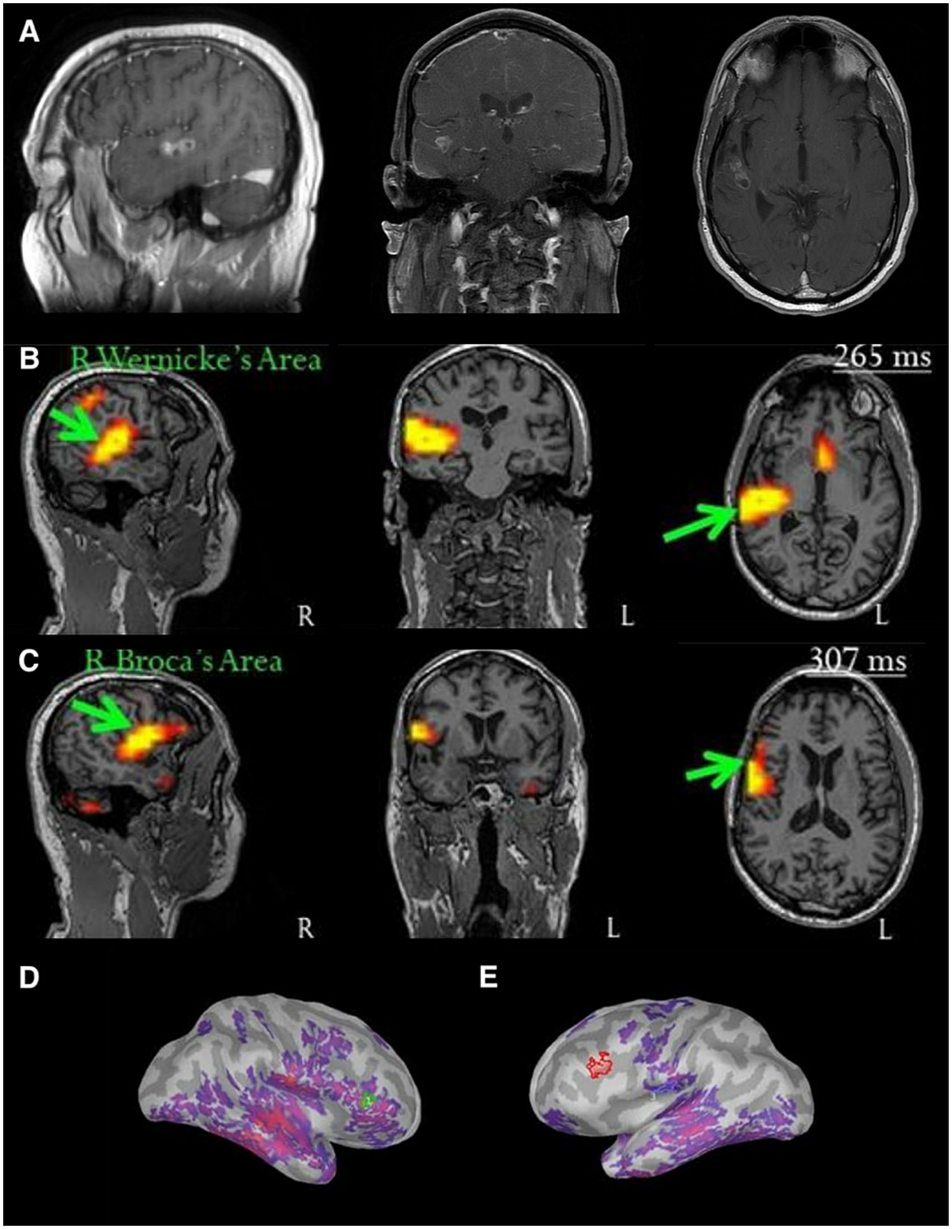
Multimodal preoperative workup for a right temporal glioblastoma. All scans are displayed according to radiologic convention. **(A)** Structural MRI demonstrating nodular enhancement near the prior resection cavity at the superior aspect of the right temporal lobe, **(B)** magnetoencephalography (MEG) demonstrating activation of the right posterior temporal lobe, consistent with Wernicke’s area, using a word comprehension paradigm, **(C)** MEG demonstrating activation of the right frontal lobe, consistent with Broca’s area, using a naming task, and **(D,E)** show images rendered from language activation patterns on MEG in the right and left hemispheres, respectively, superimposed on the patient’s structural MRI.

MRI at presentation revealed increased nodular enhancement in the right temporal lobe, measuring 2.5 × 1 cm, with elevated relative cerebral blood volume on perfusion imaging, which was indicative of disease progression (Figure 1). Neuropsychology was consulted to evaluate his language, which revealed an intermittent response latency indicative of effortful lexical retrieval. His speech and language were otherwise grossly unremarkable, including intact oral motor control, comprehension, phonological and morphological processing, grammatical construction, repetition, reading, and writing. The Edinburgh Handedness Inventory – Short Form (EHI-SF) (Veale, 2014) confirmed right-hand preference [laterality quotient (LQ) = 75], with mixed handedness for sports. He obtained a high school diploma with no known developmental delays. There was also no indication of postoperative aphasia from his prior resection based on self- and informant reports. The patient was referred for a multidisciplinary preoperative evaluation for complex brain tumor resection based on these findings.

### 2.1 Clinical data

We employed a multidisciplinary preoperative protocol that included structural and functional neuroimaging, tractography, and a neuropsychological evaluation (NPT) to preoperatively identify the language-dominant hemisphere, assess tumor proximity to eloquent areas, and evaluate the impact of the tumor on verbal versus nonverbal cognition.

### 2.2 Structural imaging

MRI is essential in brain tumor surgery, providing detailed noninvasive visualization of the lesion’s location, the surrounding structures, and the tumor’s vasculature. The patient’s preoperative MRI revealed nodular enhancement near the resection cavity in the right superior temporal lobe along the Sylvian fissure. Despite new word-finding difficulties and tumor progression in a critical area, the team was initially skeptical about the need for awake language mapping due to the tumor’s RH location, the patient’s strong right-handedness, and a prior surgery performed under general anesthesia without postoperative aphasia. However, it was decided by the neurosurgical team that additional evaluation was warranted to better localize language function.

### 2.3 Non-invasive functional mapping of language

Functional imaging techniques, such as functional MRI (fMRI) and magnetoencephalography (MEG), along with neurostimulation methods, such as navigated transcranial magnetic stimulation (nTMS), are non-invasive tools to assess functional brain activity during cognitive tasks. The selection of modality often depends on institutional resources and the clinical context.

To assess language dominance near the superior temporal lobe, two language protocols were conducted with MEG. Receptive language was evaluated utilizing a word-listening paradigm in which the patient was exposed to five target words and subsequently asked to identify them from a series of 105 written lexical (i.e., real) distractor words by raising a finger. Figure 1 shows that there was significant activation in the right posterior temporal lobe with this paradigm, which was consistent with Wernicke’s area. A two-step expressive language protocol followed, which combined object naming of simple nouns from various semantic categories (e.g., an image of a car) with written word matching (e.g., “crab” spelled in text). This paradigm was utilized to elicit language production, lexical access, and semantic integration. The results revealed activation in the RH with language production consistent with Broca’s area, as shown in Figure 1. The patient’s MEG scan revealed that both the right temporal and frontal lobes were highly eloquent for language, in addition to activation observed in the LH. This finding suggested that the patient was atypically organized with bilateral language representation.

### 2.4 Tractography

**Figure 2:**
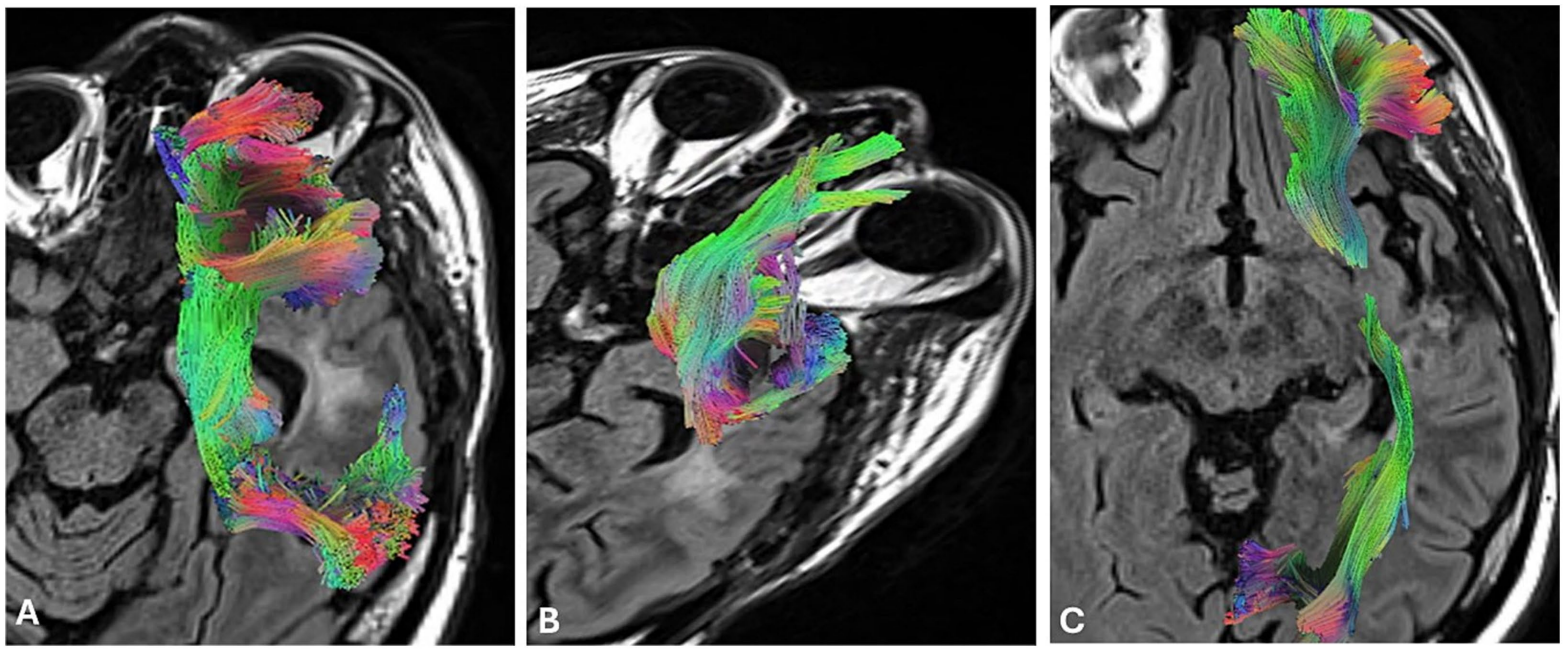
High-definition fiber tracking (HDFT) for language tracts proximal to the tumor Note: All scans are displayed in radiologic convention. **(A)** Arcuate fasciculus (AC), **(B)** uncinate fasciculus (UC), and **(C)** inferior fronto-occipital fasciculus (IFOF).

High definition-fiber tracking (HDFT) is a tractography method that allows for precise 3D visualization of white matter tracts and their cortical connections, offering neurosurgeons an anatomical roadmap to support surgical planning and avoid postoperative aphasia (Panesar et al., 2019). However, HDFT does not provide functional information and may be unreliable at times (Voets et al., 2021), such as in the presence of peritumoral edema. Neurosurgeons must often supplement HDFT with intraoperative adjuncts such as DES to accurately identify and preserve functional pathways. In this case, the patient’s HDFT (Figure 2) revealed potential edema-related distortion of the right arcuate fasciculus (AF), a tract connecting Broca’s and Wernicke’s areas—but no evidence of tumor infiltration into critical subcortical pathways. These findings supported the feasibility of a gross total resection and reduced concerns about permanent language deficits. The neurosurgical team considered whether to use cortico-cortical evoked potentials (CCEPs) under general anesthesia to assess AF function or to proceed with an awake craniotomy. Given concerns about potential involvement of the inferior fronto-occipital fasciculus (IFOF) due to the patient’s lexical retrieval difficulties, comprehensive NPT was pursued to determine intraoperative mapping (IOM) candidacy and evaluate global cognitive functioning.

### 2.5 Neuropsychological evaluation

Neuropsychology provides a translational approach to understanding the quantitative effects of a brain tumor on cognition and quality of life (QoL). Targeted test batteries quantify cognitive function based on tumor location and the functional skills essential to the patient (Morshed et al., 2021), which can help determine the proximity of the tumor to eloquent networks, the laterality of language dominance, and candidacy for IOM. The most widely utilized paradigm for IOM of language is object naming, given its ease of use in the operating room and its ability to detect multiple linguistic processes (Martín-Monzón et al., 2022). Patients are trained on selected paradigms at baseline to optimize compliance during IOM.

**Figure 3:**
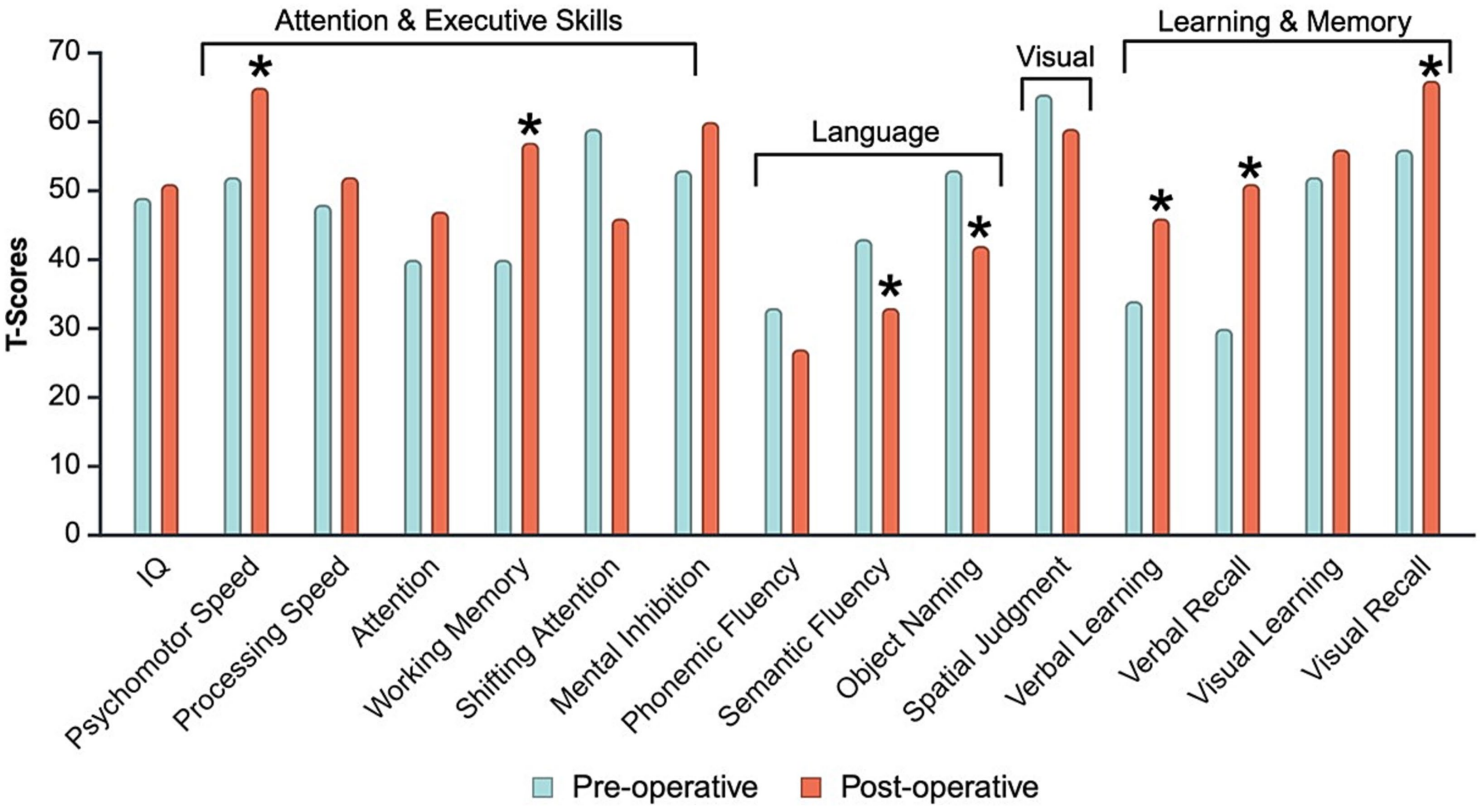
Preoperative and postoperative neuropsychological test results.*Denotes a one-standard-deviation difference between preoperative and postoperative data. Values are represented in T-scores (A T-score = 50 is equivalent to the 50th percentile (average range) on the normal curve and has a standard deviation of 10).

An NPT evaluation indicated occasional speech hesitations suggestive of mild, effortful lexical retrieval; however, spontaneous speech and language were otherwise intact. Quick Aphasia Battery (QAB) (Wilson et al., 2018) performance was largely error-free, aside from a single object naming failure and one minor repetition error on a complex phrase, yielding a technically normal score (9.86). No semantic, phonological, grammatical, or syntactic errors were observed. Writing ability, screened using the bedside Western Aphasia Battery – Revised (WAB-R) (Kertesz, 2006), was similarly intact. Generally, the NPT indicated globally intact cognitive functioning, aside from mild impairment in higher-level executive-linguistic skills and bilateral fine motor skills. See Figure 3 for performance across NPT, and Supplementary Table 1 for the specific NPT utilized for the evaluation. The NPT results supported language representation in the RH, as there was a greater decline in verbal compared to non-verbal functions and possible involvement of semantic language networks given the presence of mild anomia (Figure 3).

**Figure 4:**
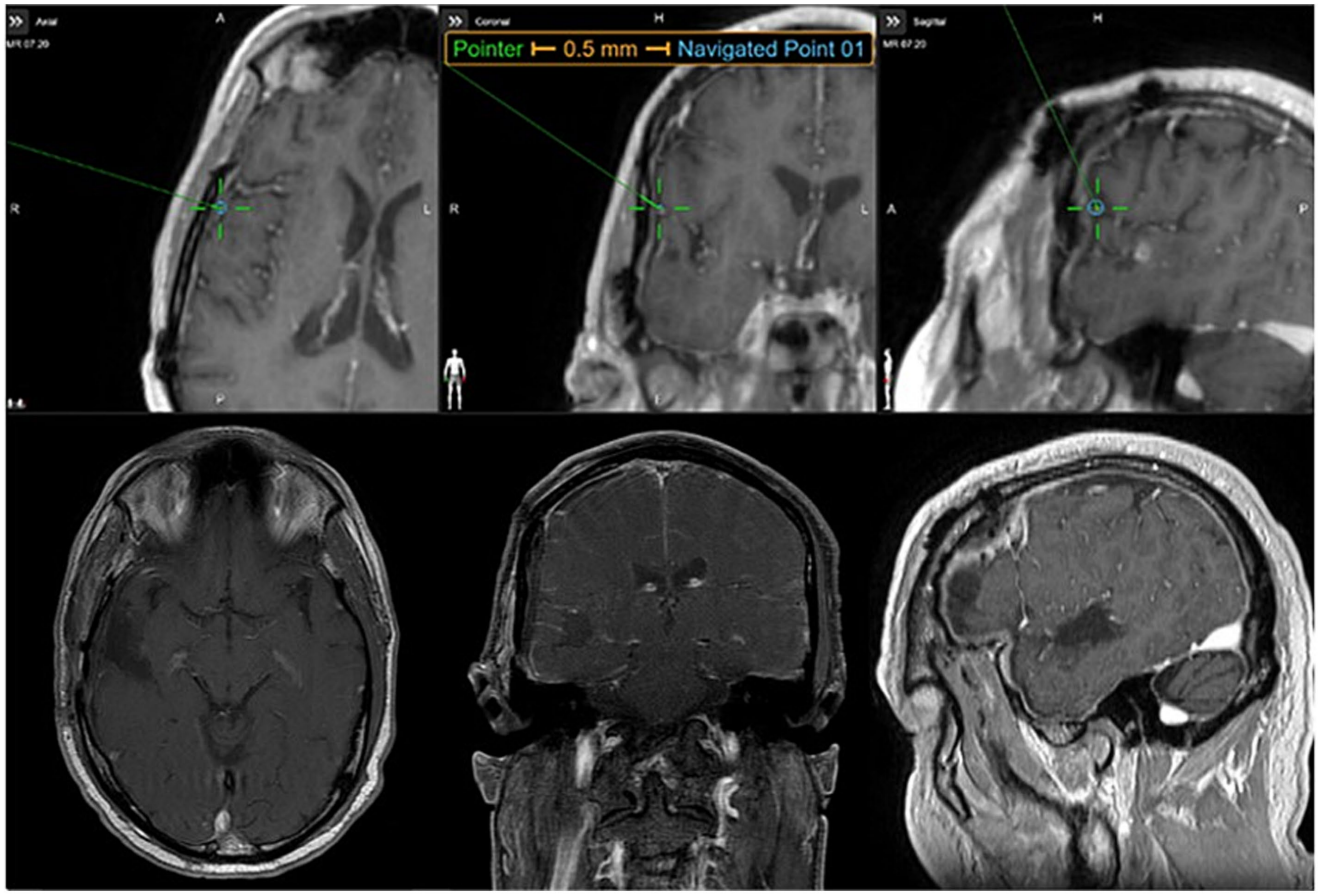
Intraoperative mapping with positive language findings in the right hemisphere and postoperative MRI. All scans are displayed in radiologic convention. **(A)** Positive language findings in the right frontal lobe when the patient exhibited speech arrest at 10 mA during direct electrical stimulation (DES) and **(B)** postoperative MRI showing a gross total resection of the recurrent glioblastoma.

To establish a baseline for IOM, the patient completed several language tasks to assess feasibility in the operating room. Assessing naming, reading, and spontaneous speech are core paradigms for language mapping and engage multiple linguistic processes (Vilasboas et al., 2017; Rofes et al., 2015). Noun-naming was prioritized over verb comprehension and production, given the posterior temporal lobe location of the tumor (Rofes and Mahon, 2021). In addition, we monitored action naming and other language functions through spontaneous speech. The patient performed adequately (Morshed et al., 2021) on baseline testing for IOM: picture naming (76/90; 84% correct), word reading (40/40; 100% correct), number reading (30/30; 100% correct), and auditory comprehension (84/89; 94% correct). We utilized a 25% error rate to determine the viability of a task to be utilized intraoperatively (Rofes and Mahon, 2021). The patient and the neurosurgical team made an informed decision that, given the aggressive nature of the tumor, preservation of speech was the primary skill to maintain, and that nonverbal skills were not a priority.

## 3 Results

The neurosurgical team utilized the multidisciplinary evaluation to render a decision about the localization of language function and whether this right-handed patient would benefit from an awake procedure for a recurrent RH tumor. Converging evidence, including the patient’s clinical presentation, MRI, and HDFT showing the tumor was proximal to the presumed language networks, MEG demonstrating bilateral language activation, and NPT revealing greater deficits in verbal versus non-verbal areas, led to the decision that an awake procedure with IOM was warranted. The patient was brought to the operating room for an asleep-awake-asleep craniotomy. IOM was conducted in multidisciplinary coordination between the neuropsychologist, neurophysiologist, and neurosurgeon.

### 3.1 Intraoperative mapping

We performed cognitive mapping utilizing an innovative, tablet-based dual iPad application (Sabsevitz et al., 2020) for the administration of neuropsychological paradigms. The application features sophisticated functions to streamline mapping, including selecting only stimuli that were answered correctly preoperatively, monitoring reaction time, pairing stimulus presentation with an auditory cue for stimulation, and toggling between cognitive tasks. DES with a bipolar electrode was performed to map the cortical surface. Positive sensorimotor and speech stimulation was obtained at increasing amplitudes, starting at 4 mA and progressing up to 10 mA in the posterior portion of the inferior frontal gyrus and the posterior portion of the superior temporal gyrus, guiding the corticectomy to avoid eloquent areas. An error was considered a positive finding if it could be replicated two to three times, and positive findings were mapped anatomically using neuronavigation. Sites were mapped with DES until either a positive finding was observed or after discharges were observed on EEG. A positive finding was always followed by no stimulation to ensure the patient returned to baseline mental status prior to proceeding with further DES.

Our patient was administered a task of object naming for the mapping portion of the surgery. Object naming was selected for mapping in this RH case due to its demand on multiple language processes (i.e., semantic and phonemic processing), its demand on visual perceptual recognition, and its ease of use in the operating room. The presence of a neuropsychologist during IOM improves the delivery of standardized administration of cognitive tasks, the detection of errors, and the ability to categorize those errors to inform which neural networks were affected.

The IOM yielded two positive sites that were reproducible. The neurosurgeon approached DES by starting at the inferior frontal gyrus, an area of presumed eloquence based on preoperative planning. In this area, the patient demonstrated no positive findings until DES was increased to 8 mA. At this threshold, the patient was presented with four object-naming trials, which yielded speech arrest on three of the four trials, confirming eloquence and Broca’s area. The neurosurgeon then mapped the superior temporal gyrus using DES. There were no positive findings across approximately 10 DES trials until reaching 10 mA. The patient committed two errors while mapping the superior temporal lobe: a mixed semantic paraphasia (“onion” for ‘tomato’) and a non-response to a picture of ‘cake’ in the same location. These items were correctly named at baseline and without stimulation. In addition to these two errors, the patient was presented with four other trials at 10 mA, and two of these trials yielded response latency; however, these findings were difficult to interpret as true positives versus non-compliance in the intraoperative setting. Given the two unequivocal positive responses and repeatable latency in lexical retrieval (Moritz-Gasser et al., 2012), the site was classified as eloquent. There was no indication of afterdischarges on EEG during DES in either region. The positive findings during mapping indicated eloquence in both the right frontal and superior temporal lobes, confirming language representation in the RH, consistent with the dorsal and ventral language streams typically expected in the LH for the majority of individuals. Once the remainder of the cortical surface was mapped, the neurosurgical team proceeded with the resection.

Neuromonitoring of mental status and function was performed continuously throughout the resection using the paradigms administered preoperatively (i.e., picture naming, word reading, number reading, and auditory comprehension), and engaging the patient in various aspects of spontaneous speech (e.g., proper naming, counting, and repetition). The patient had normal speech perception and production throughout resection and neuromonitoring. Under microscopic magnification, part of the right temporal operculum was removed, revealing the insula. Tumor tissue, which was 5-ALA positive, was visualized posterior to the insula, and a frozen sample confirmed recurrent glioma. The tumor was resected using 5-ALA guidance, while preserving insular M2 branches. No complications were encountered, and the patient demonstrated intact speech and language at the conclusion of the awake phase of the surgery prior to restarting general anesthesia for the completion of surgery.

### 3.2 Follow-up outcomes

The patient tolerated the procedure well, and his postoperative course was unremarkable, except for mild dysarthria upon waking from general anesthesia, which significantly improved within 48 h of surgery. Postoperative MRI revealed gross-total resection of the tumor with no abnormal enhancement indicative of residual disease. Pathology results were consistent with recurrent glioblastoma (MGMT promoter methylated, TERT mutation positive).

Postoperative neuropsychological testing took place 3 months after his surgery. We aim to conduct postoperative NPT within 3–6 weeks following surgery, ideally before the initiation of adjuvant therapies; this timeframe balances the need for surgical recovery with the goal of establishing a stable cognitive baseline before initiating cancer treatments that carry a risk of affecting cognitive function (Wefel et al., 2011). The patient’s NPT had to be coordinated with neuro-oncology, and he had started temozolomide at the time of his postoperative NPT. Comparison of preoperative to postoperative neuropsychological testing indicated a mild decline in semantic fluency and object naming, but significant improvements in processing speed, working memory, and aspects of learning and memory. The decline in language-based skills further confirms the presence of language eloquence in the RH. We did not observe typical signs of cognitive impairment associated with systemic cancer treatment (i.e., “chemobrain”) (Wefel et al., 2011), which was congruent with the patient’s subjective report.

## 4 Discussion

Complex brain tumor resection requires balancing maximal tumor removal with the preservation of language and other eloquent functions. We report a novel case of a 58-year-old, strongly right-handed, monolingual English-speaking male with recurrent right temporal glioblastoma and new onset mild word-finding difficulty, who was ultimately found to have RH language representation. He underwent an awake craniotomy with DES, which confirmed language representation in the right frontal and temporal lobes.

Historically, language mapping with DES for RH tumor is an uncommon practice given the predominance of LH language dominance (Young et al., 2021; Knecht et al., 2003). There is increasing recognition that language is not strictly left-lateralized and may be more distributed than previously believed (Vassal et al., 2010), particularly in patients with prior neurological insults, such as tumors or previous surgeries (Pasquini et al., 2022). These findings underscore the need for individualized functional mapping and broader outcome research in RH tumors, along with increased attention to higher-order cognitive domains beyond traditional language and sensorimotor networks. This case highlights the notion that a “typical” functional organization does not apply universally across patients, and thorough perioperative evaluation is necessary to optimize neurosurgical outcomes in complex brain tumors. Incorporating advanced imaging modalities with NPT may help identify atypical functional localization preoperatively, guide intraoperative decision-making, and better predict cognitive outcomes across diverse clinical presentations.

While the majority of research on language outcomes following IOM during awake craniotomy focuses on LH pathology, our postoperative NPT for this RH tumor revealed comparable postoperative language effects. At 3-month follow-up, our patient demonstrated a significant decline in object naming and semantic fluency, despite preserved object naming and spontaneous speech intraoperatively. These results are consistent with findings in LH tumors (Norrelgen et al., 2020; Satoer et al., 2018), suggesting that IOM using object naming may not sufficiently assess the complexity of language (Papagno et al., 2012). In addition to language declines, as anticipated, our patient also exhibited a significant decline in visuospatial and executive skills, reinforcing the RH’s dual role in both language and spatial processing for this patient. There is growing recognition of the need to routinely monitor non-language cognitive functions (e.g., visuospatial judgment and executive functioning) (Sabsevitz et al., 2020; Ekert et al., 2024), though there remains debate about whether these domains are critical enough to influence surgical decision-making, particularly when weighing the onco-functional balance (Duffau and Mandonnet, 2013). While monitoring of global cognitive function would be ideal, intraoperative constraints limit the number of functions that can be mapped, requiring prioritization based on real-world relevance and patient goals (Moritz-Gasser et al., 2012; Duffau and Mandonnet, 2013).

Future research should identify which intraoperative tasks best predict functional outcomes and which are most relevant to patients’ daily lives. Importantly, the majority of studies to date focus on LH lesions; little is known about the relationship between intraoperative language monitoring and postoperative outcomes for RH tumors, particularly in patients with atypical or mixed language dominance. A broader investigation into RH mapping and postoperative recovery trajectories is needed to inform evidence-based surgical decision-making and improve individualized care.

## 5 Conclusion

This case underscores the importance of individualized surgical planning for complex brain tumors, regardless of tumor laterality. Despite this patient’s strong right-handedness and absence of known risk factors for atypical language lateralization, comprehensive multimodal preoperative evaluation revealed RH language dominance confirmed with DES. These findings challenge the dogma that awake craniotomy and language mapping are primarily indicated for LH lesions and highlight the value of systematic preoperative assessment.

## Data Availability

The raw data supporting the conclusions of this article will be made available by the authors, without undue reservation.
