# Supplementary material for: Case Report: A personalized model of care for surgical resection of complex brain tumor with atypical language dominance
Source: Front Neurosci. 2025 Jul 24;19:1587594. doi: 10.3389/fnins.2025.1587594 (PMC12328450; doi:10.3389/fnins.2025.1587594)
Supplement: Supplementary file 1 [file Table_1.docx]

Supplemental Table 1. Neuropsychological Tests Utilized for Pre- and Post-operative Evaluation

| Neuropsychological Test | Pre-operative Testing | Post-operative Testing |
| --- | --- | --- |
| TOPF | 49 | 57 |
| TMT A | 52 | 65* |
| TMT B | 59 | 46* |
| WAIS-IV Digit Span Forward | 40 | 47 |
| WAIS-IV Digit Span Backward | 40 | 57* |
| SDMT | 48 | 52 |
| D-KEFS Color-Word Interference Inhibition | 54 | 60 |
| D-KEFS Letter Fluency | 34 | 28 (Alternative Form) |
| D-KEFS Category Fluency | 43 | 33* (Alternative Form) |
| NAB Naming | 53 (Form 1) | 42* (Form 2) |
| JoLO-SF | 64 | 59 |
| HVLT-R Total Recall | 34 (Form 1) | 46 (Form 4)* |
| HVLT-R Delayed Recall | 30 (Form 1) | 51 (Form 4)* |
| BVMT-R Total Recall | 52 (Form 1) | 56 (Form 4) |
| BVMT-R Delayed Recall | 56 (Form 1) | 66 (Form 4)* |
| Grooved Pegboard Test (Right hand) | 31 | 35 |
| Grooved Pegboard Test (Left hand) | 31 | 35 |

TOPF (Test of Premorbid Functioning); TMT A&B (Trail Making Test – A & B); WAIS-IV (Wechsler Adult Intelligence Scale – IV); SDMT (Symbol Digit Modality Test); D-KEFS (Delis-Kaplan Executive Function System); NAB (Neuropsychological Assessment Battery); JoLO-SF (Judgment of Line Orientation – Short-Form); HVLT-R (Hopkins Verbal Learning Test – Revised); BVMT-R (Brief Visuospatial Memory Test – Revised).
